# Supplementary material for: Exploring Contemporary Data on Lipid-Lowering Therapy Prescribing in Patients Following Discharge for Atherosclerotic Cardiovascular Disease in the South of Italy
Source: J Clin Med. 2022 Jul 26;11(15):4344. doi: 10.3390/jcm11154344 (PMC9369296; doi:10.3390/jcm11154344)
Supplement: Supplementary file 1 [file jcm-11-04344-s001.zip › jcm-1829090-supplementary.pdf]

Table S1. ASCVD conditions identified by the International Classification of Diseases 9th Revision, Clinical Modification (ICD-9-CM), diagnosis and procedures codes.

	ICD-9 Diagnosis Codes [1]	ICD-9-CM Procedures Codes [1]
**ASCVD CONDITION**		
ACS	410.xx, 412.xx	
Stable angina	413.xx	
Unstable angina	411.xx	
PAD	250.7x, 441.xx, 42.xx, 443.xx, 444.xx, 447.1	38.13, 38.14, 38.16, 38.18, 39.25, 39.29, 39.50, 39.90, 99.10
Stroke/TIA	433xx–435xx	38.11, 38.12
Revascularization procedures [2]	V45.81, V45.82	36.01, 36.02, 36.03, 36.05, 36.06, 36.07, 0.66, 36.1x, 36.04, 36.2, 36.3, 36.9, 37.8x

[1] Diagnosis or procedure codes from 1 January 2019–30 June 2020 recorded in hospital discharge records (HDRs). [2] Revascularization procedures including percutaneous coronary intervention (PCI), coronary artery bypass surgery (CABG) or other revascularization procedures. Abbreviations: ACS, Acute Coronary Syndrome; ASCVD, Atherosclerotic Cardiovascular Disease; CABG, Coronary Artery Bypass Surgery; PAD, Peripheral Arterial Disease; PCI, Percutaneous Coronary Intervention; TIA, Transient Ischemic Attack.

Table S2. Comorbidities identified by the International Classification of Diseases 9th Revision, Clinical Modification (ICD-9-CM) codes, the Anatomical Therapeutic Chemical classification (ATC-codes) and exemption codes.

	ICD-9 Codes [1]	ATC-Codes [2]	Exemption Codes
**BASELINE COMORBIDITIES**			
Hypertension	362.11, 401x–405x, 437.2		
Diabetes	250xx, 357.2, 362.0x, 366.41	A10*2	013x
Chronic ischemic heart disease	414.xx		
Congestive heart failure	428.x, 785.5x, 402.01, 402.11, 402.91, 404.01, 404.03, 404.11, 404.13, 404.91, 404.93		
Chronic kidney disease	585.xx		
Other cerebrovascular disease	430xx–432xx, 436xx–438xx		
Other heart disease	420–421, 423–424, 429, 785.0–785.3, V42.2, V43.3		
Liver disease	572.xx–573.xx, 864.xx,		
Familial hypercholesterolemia	272.0–3x		025x

[1] Diagnosis codes within 8 years before data index date recorded in hospital discharge records (HDRs). [2] Filled prescription recorded in the outpatient pharmaceutical databases within 1 year before index date the hospital discharge for ASCVD during the period between 1 January 2019 and 30 June 2020 (enrollment period).

Table S3. Demographic and clinical characteristics for ASCVD patients stratified by type of lipid lowering therapy.

	Low-Intensity Statin [1]	Moderate-Intensity Statin [1]	High-Intensity Statin [1]	PCSK9i [2]
	N = 90	N = 2441	N = 2339	N = 64
	n (%)	n (%)	n (%)	n (%)
**Gender**				
Male	33 (36.7)	1142 (59.1)	1699 (72.6)	50 (78.1)
Female	57 (63.3)	999 (40.9)	640 (27.4)	14 (21.9)
**Age (years)**				
18–54	3 (3.3)	194 (7.9)	344 (14.7)	20 (31.3)
55–64	10 (11.1)	416 (17.0)	574 (24.5)	26 (40.6)

65–74	29 (32.2)	755 (30.9)	724 (31.0)	14 (21.9)
≥75	48 (53.3)	1076 (44.1)	697 (29.8)	4 (6.3)
**ASCVD condition**				
Stroke/TIA	29 (32.2)	949 (38.9)	317 (13.6)	1 (1.6)
Procedures [3]	23 (25.6)	548 (22.4)	431 (18.4)	12 (18.8)
ACS [4]	13 (14.4)	258 (10.6)	535 (22.9)	15 (23.4)
ACS and procedures [3]	8 (8.9)	191 (7.8)	596 (25.5)	18 (28.1)
PAD	5 (5.6)	227 (9.3)	115 (4.9)	
Stable angina [4]	7 (7.8)	156 (6.4)	115 (4.9)	4 (6.3)
Unstable angina [4]	4 (4.4)	76 (3.1)	120 (5.1)	3 (4.7)
Unstable angina and procedures [3]	0	17 (0.7)	68 (2.9)	3 (4.7)
Stable angina and procedures [3]	1 (1.1)	19 (0.8)	42 (1.8)	8 (12.5)
**Comorbidities**				
Hypertension	63 (70.0)	1607 (65.8)	1317 (56.3)	37 (57.8)
Diabetes	44 (48.9)	1150 (47.1)	972 (41.6)	16 (25.0)
Chronic ischemic heart disease	29 (32.2)	800 (32.8)	1047 (44.8)	33 (51.6)
Congestive heart failure	19 (21.1)	444 (18.2)	331 (14.2)	7 (10.9)
Chronic kidney disease	17 (18.9)	460 (18.8)	268 (11.5)	4 (6.3)
Other cerebrovascular disease	22 (24.4)	545 (22.3)	243 (10.4)	4 (6.3)
Other heart disease	8 (8.9)	291 (11.9)	221 (9.4)	5 (7.8)
Liver disease	2 (2.2)	31 (1.3)	16 (0.7)	0
Familial hypercholesterolemia	0	49 (2.0)	33 (1.4)	4 (6.3)
**Previous ASCVD event**	14 (15.6)	495 (20.3)	534 (22.8)	18 (28.1)

[1] With and without ezetimibe. [2] With and without ezetimibe and/or statin therapies. [3] Procedures including percutaneous coronary intervention, coronary artery bypass surgery, or other revascularization procedures. [4] Without procedures. Abbreviations: ACS, acute coronary syndrome; ASCVD, atherosclerotic cardiovascular disease; PAD, peripheral arterial disease; TIA, transient ischemic attack; PCSK9i, proprotein convertase subtilisin kexin 9 inhibitors. High-intensity statin: Atorvastatin 40–80 mg, Rosuvastatin 20–40 mg. Moderate-intensity statin: Atorvastatin 10–20 mg, Fluvastatin 80 mg, Lovastatin 40 mg, Pitavastatin 2–4 mg, Pravastatin 40–80 mg, Rosuvastatin 5–10 mg, Simvastatin 20–40 mg. Low-intensity statin: Fluvastatin 20–40 mg, Lovastatin 20 mg, Pitavastatin 1 mg, Pravastatin 10–20 mg, Simvastatin 10 mg.
